# Soluble Urokinase Plasminogen Activator Receptor as a Marker for Use of Antidepressants

**DOI:** 10.1371/journal.pone.0110555

**Published:** 2014-10-20

**Authors:** Eva Haastrup, Katrine Grau, Jesper Eugen-Olsen, Christian Thorball, Lars Vedel Kessing, Henrik Ullum

**Affiliations:** 1 Department of Clinical Immunology, the Blood bank, Rigshospitalet, University Hospital of Copenhagen, Copenhagen, Denmark; 2 Department of Epidemiology Research, Statens Serum Institut, Copenhagen, Denmark; 3 Clinical Research Centre, Hvidovre Hospital, University Hospital of Copenhagen, Copenhagen, Denmark; 4 Psychiatric Centre, Rigshospitalet, University Hospital of Copenhagen, Copenhagen, Denmark; Charite Universitätsmedizin Berlin, Germany

## Abstract

**Objectives:**

Inflammation is involved in the pathogenesis of depression. A few cross-sectional population-based studies have found that depression is associated with increased levels of inflammatory markers. Soluble urokinase plasminogen activation receptor (suPAR) is known to be a stable marker for inflammation. We investigated the bidirectional association between suPAR levels and use of antidepressants.

**Methods:**

suPAR level was measured in 9305 blood donors and analysed in relation to 5-years follow-up data on purchase of antidepressants and hospital diagnoses of depression from a nationwide Danish register.

**Results:**

For men and women without prior use of antidepressants we found a significantly higher risk for incident use of antidepressants with higher suPAR values. For men, the risk of first use of antidepressants increased by 72% from the 1^st^ to the 4^th^ quartile (HR = 1.72, 95% CI: 1.11–2.69). For women, it increased by 108% from the 1^st^ to the 4^th^ quartile (HR = 2.08, 95% CI: 1.45–2.98). Previous use of antidepressants was also significantly associated with higher suPAR levels (p = 0.002).

**Conclusions:**

High suPAR levels are associated with an increased risk for both previous and future use of antidepressants in healthy men and women. High suPAR are also associated with increased risk for a hospital diagnosis of depression.

## Introduction

In the last decade, it has become clear that there is a biological link between inflammation and depression. In patients with depression, several studies have demonstrated increased expression of proinflammatory cytokines, chemokines, acute phase reactants, and adhesion molecules compared to non-depressed controls [1]–[9]. However, there is ongoing debate on whether elevated systemic inflammation is a biological mechanism leading to depression.

A few population-based studies have investigated the relationship between C-reactive protein (CRP) and de novo depression. In a study including 644 women with no prior history of depression, of whom 48 women developed depression during the 5827 person-years of follow-up, CRP was found to be an independent predictor of depressive disorder, supporting an aetiological role for inflammatory activity in the pathophysiology of depression [10]. Two larger population-based studies have investigated the direction of the depression-inflammation relationship. The SWAN study of 3292 pre- and postmenopausal women found that over a five-year observation period, higher depression scores were related to higher levels of fibrinogen and plasminogen activation inhibitor type one (PAI-1) but not CRP [11]. This was on the other hand reported by Deverts et al [12] in the CARDIA study, where depressive symptoms at baseline were positively correlated with CRP levels measured at 5-years follow-up. No association was found between CRP levels at the beginning of the observation period and subsequent depressive symptoms in any of these studies. This was however the main finding in a large study by Wium-Andersen et al [13].

The lack of consensus with regard to CRP and development of depression may in part be due to the high biological variation of CRP (hsCRP). In 38 healthy blood donors with 6 determinations over 22 days a biological variation of hsCRP of 50% was found [14]. Other studies have shown similar biological variation, ranging from 30–63% [15], [16].

SuPAR is a protein that is measurable in the circulating blood of all individuals. In contrast to most pro-inflammatory and acute response biomarkers, circadian changes in plasma suPAR is minimal, and in vitro stability is also high [17]–[19].

Elevated levels of suPAR are associated with immune activation, inflammation and a negative outcome in patients with symptoms of infection (recently reviewed in “Usefulness of suPAR” as a biological marker in patients with systemic inflammation or infection: a systematic review [20]).

Furthermore, in an observational prospective Danish cohort (MONICA) consisting of healthy individuals, plasma suPAR levels were associated to the development of cancer, cardiovascular disease, type-2 diabetes and mortality during 12-years follow-up [21]. SuPAR and CRP seems to reflect different aspects of inflammation. A recent study suggested that CRP is associated with anthropometric measures of inflammation, whereas suPAR is linked to cellular and vascular inflammatory processes [22].

To test the hypothesis of inflammation and development of depression, we investigated whether the biomarker suPAR was associated with an increased risk of developing depression in a large cohort of Danish blood donors. As a proxy for depression, we used purchase of antidepressants, supplemented with a discharge diagnosis of depression.

Aim of the study was to test the hypothesis, that higher levels of circulating suPAR in healthy individuals are associated with an increased risk for future use of antidepressants or a hospital diagnosis of depression. From both analyses were donors with a pre-history of antidepressant use or a previous hospital diagnosis of depression excluded. We further tested the hypothesis that prior use of antidepressants was associated with higher levels of suPAR.

## Methods and Materials

### Ethical considerations

This study was approved by the local Medical Scientific Ethics Committee of Denmark (H-KF 01283058). All participants provided written informed consent. This study was conducted in accordance with the Declaration of Helsinki.

### The cohort

Blood donors, who donated blood to the Copenhagen City Blood bank (former H:S Blood bank) during a three month period from the 14^th^ of January to the 24^th^ of April 2006 were invited to participate in the study. The inclusion criterion was fulfilment of the Danish criteria for blood donation (i.e. self-reported good health and lack of behavioural risks for transfusion transmissible infections). All donors were asked about medication usage. Usage of antidepressive medicine within the last two weeks prior to visiting the blood bank was an exclusion criterion for donation and thus participation. Informed written consent was obtained from 11971 donors. Blood samples were obtained from a look-back repository where samples were available for 9312 of these donors.

### suPAR measurement

All blood samples were collected in 7-ml blood EDTA anticoagulated tubes with stabilising gel (Becton Dickenson, Glostrup, Denmark). Blood samples were centrifuged to separate the plasma from the cells within 1 hour and frozen within 6 hours.

Plasma suPAR was measured at Hvidovre hospital with the suPARnostic ELISA kit (ViroGates Copenhagen, Denmark) according to the manufacturer instructions. The samples were measured in singlets and the interassay variation of a control plasma sample run on all plates was <10%. The kit standard curve was validated to measure suPAR levels between 0.6 and 22 ng/ml.

### Danish register data

Data on purchase of antidepressive medicine and other prescription medicines from the Danish register of Medical Products [23] was linked to study participants via their central personal registration number (CPR-number). The CPR-number is a unique number ascribed to each Danish citizen. The Medical Product Register Statistics contains information on all medical prescriptions purchased in Denmark from the first of January 1995 to 31^st^ of December 2010. In Denmark, all antidepressive or antipsychotic medications are prescribed by doctors and can only be purchased at pharmacies. The sale of prescribed medication from the pharmacies is registered with the name of the drug, Anatomical Therapeutical Chemical code (ATC code), dose, CPR-number of the patient and the identity of the doctor prescribing the medicine.

From the Danish Patient Registry we obtained data on hospitalization with depression. Diagnoses were based on the International Statistical Classification of Disease (ISCD).

#### Cases

Case persons were persons who filled prescriptions for antidepressive medication (any ATC code beginning with N06A registrated in the Register of Medicinal Products Statistics) or had a hospital discharge diagnose with the codes F.32 or F.33.

#### Exclusion

When analysing incident depression, donors who purchased antidepressants before the time of suPAR measurement were excluded.

Similarly were donors with a hospital discharge diagnosis of depression before the time of suPAR measurement, excluded from the analysis of incident hospitalization.

In the following text, references to the purchase and use of antidepressants will be used interchangeably. Other medications were all prescription medications purchased other than antidepressants.

### Statistical analysis

#### Analysing the incident use of antidepressants

As a proxy for incident depression, we investigated the first use of antidepressants between suPAR measurement and the 31^st^ of December 2010 in participants who never purchased antidepressants before suPAR the measurement, according to the register.

The incident use of antidepressants after suPAR measurement was illustrated according to sex-specific suPAR level quartiles by Kaplan-Meier curves and compared using a log-rank test. Cox regression analyses stratified by sex, with age as the underlying timescale, were further used to assess the effect of suPAR level on the subsequent incident use of antidepressants. The interaction between sex and suPAR in relation to time of first purchase of antidepressant medication was investigated in a model with suPAR sex and a sex-suPAR level interaction term. Analyses were conducted using the log2-transformed suPAR level as a continuous variable and with quartiles of suPAR levels. Analyses were repeated while also adjusting for the purchase of prescription medication other than antidepressants as a time-dependent variable.

The same analysis was used to examine if there was an association between the suPAR level of the donors and a hospital discharge diagnosis of depression but only with suPAR as continuous variable adjusted for sex.

#### Analysing prior use of antidepressants

We tested the hypothesis that a prior purchase of antidepressants was associated with increased suPAR levels. The effect of prior purchase of antidepressants on the log2-transformed suPAR level data was estimated using linear regression models adjusted for age (continuous linear variable). Separate analyses were conducted for women and men. The purchase of antidepressants was analysed as a dichotomous variable (none/any) and in categories of time from last purchase to suPAR measurement (none, less than 6 months, 6 to 12 months and 12 month or more).

## Results

### Descriptive statistics

A total of 9312 individuals with an available blood sample had their suPAR level measured. Seven blood samples were excluded due to outlying low values. Thus, the cohort consisted of 9305 individuals, including 4464 women and 4841 men. There were more women than men in the younger age group (18–30 years of age) while there were more men than women in all other age groups. The median age for women was 36 years (quartiles: 27–47) and the median age for men was 39.9 years (quartiles: 30–49).

The median suPAR values were 2.54 ng/ml (range 0.6–19.4) for women and 2.22 ng/ml (range 0.6–15.4) for men. suPAR levels increased with age in both men and women. In women there was an increase between the 18–30 and 31–40 age groups from 2.43 ng ml^−1^ to 2.58 ng/ml after which the level increased slowly to 2.71 ng/ml. For men the median suPAR value increased more evenly across the age groups from 2.08 ng/ml to 2.44 ng/ml (Table 1).

**Table 1:** Soluble urokinase plasminogen activator receptor (suPAR) (ng/ml) in blood donors according to sex and age.

	Age	N	Median	Lower 25th percentile	Upper 25th percentile	Range
**Men**	18–30	1257	2.08	1.76	2.44	0.61–15.41
	31–40	1416	2.17	1.85	2.57	0.64–13.83
	41–50	1112	2.25	1.90	2.69	0.70–11.10
	51–65	1056	2.44	2.02	2.90	0.64–12.27
**Women**	18–30	1672	2.43	2.05	2.79	0.77–6.84
	31–40	1028	2.58	2.16	3.04	0.60–8.04
	41–50	978	2.62	2.18	3.12	0.88–8.64
	51–65	786	2.71	2.25	3.23	0.67–19.38

### Data from the Danish register of Medical Products

During the observation period from the first of January 1995 to the 31^st^ of December 2010 the 9305 donors made 387.837 purchases of prescription medications, of which 6347 were for antidepressants (ATC code ‘N06A’) made by 856 individuals. There were 445 donors who purchased antidepressants before suPAR measurement and 411 donors who made their first recorded purchase after suPAR measurement.

#### suPAR measurement and the subsequent first use of antidepressants

**Figure 1:**
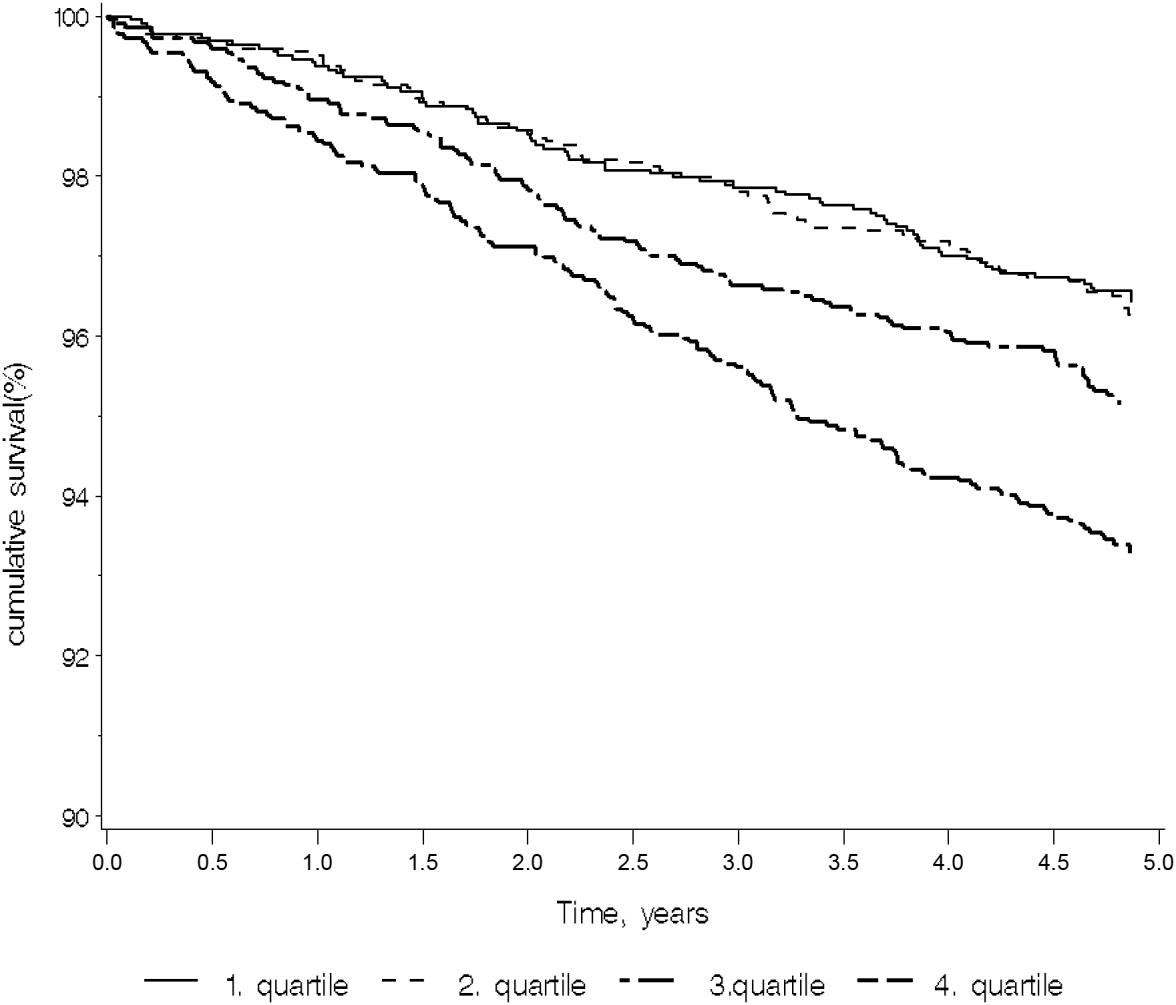
Kaplan-Meier plot with antidepressants as outcome and the suPAR level quartiles as explanatory variables (n = 9305). The 4^th^ quartile is the highest values. Log-rank test: p<0.0001.

The 8860 donors who had previously never purchased antidepressants at the time of suPAR measurement were included in the analysis evaluating the correlation between suPAR and the subsequent first use of antidepressants. In the 5-year period from suPAR measurement to the end of follow-up in December 2010, 411 donors (247 women and 164 men) purchased antidepressant medication. The majority made less than four purchases in the period. There was a statistically significant difference in the incidence of the first use of antidepressants among the suPAR level quartiles (p<0.0001), Figure 1). The first use of antidepressants occurred significantly more often for individuals in the 4^th^ suPAR level quartiles than for those in the 1^st^, 2^nd^ and 3^rd^ quartile. The association between suPAR level and first use of antidepressants persisted when stratifying by sex and adjusting for age (Table 2). Both men and women in the 4^th^ suPAR level quartile had increased rates of first use of antidepressants compared with the rates for individuals in the lowest suPAR level quartile (Table 2). For men, the risk of first use of antidepressants increased by 72% from the 1^st^ to the 4^th^ quartile (HR = 1.71, 95% CI: 1.11–2.69). For women, the risk of using antidepressants for the first time increased by 108% from the 1^st^ to the 4^th^ quartile (HR = 2.08, 95% CI: 1.45–2.98).

**Table 2:** Risk^1^ of incident use of antidepressants according to the suPAR level.

		Analysis withoutother medication			Analysis withother medication		
	suPARQuartiles	HR	95% CI	P-value	HR	95% CI	P-value
**Men, n = 4671**	q2	1.04	0.64, 1.69	0.86	1.05	0.65, 1.70	0.86
**Events = 164**	q3	1.34	0.89, 2.21	0.15	1.40	0.89, 2.21	0.15
	q4	1.72	1.11, 2.69	0.02	1.73	1.11, 2.70	0.02
	Other				1.24	0.90, 1.72	0.18
**Women,** **n = 4189**	q2	1.04	0.69, 1.55	0.87	1.03	0.69, 1.55	0.87
**Events = 247**	q3	1.37	0.94, 2.01	0.11	1.37	0.93, 2.0	0.11
	q4	2.08	1.45, 2.98	<0.0001	2.08	1.45, 2.98	0.0001
	Other				0.76	0.56, 1.03	0.08

1 Estimates are hazard ratios from Cox regression analyses stratified by sex and with age as the underlying time scale. Purchase of antidepressants is used as proxy for depression. The analysis is performed both with and without adjustment for other medication.

The purchase of prescriptive medication other than antidepressants was not associated with an increased incidence of first antidepressant usage in men or women and the inclusion of other medication did not change the association between the suPAR level and the first use of antidepressants (Table 2). Testing for an interaction between sex and suPAR levels in relation to the first use of antidepressants revealed no statistically significant interaction (p = 0.42).

Analysis of data from the Danish Patient Register of hospitalization found an association between the suPAR levels and a hospital discharge diagnosis of depression (table 3). A higher suPAR levels was associated with a 28% increased risk for a hospital discharge diagnosis of depression (p = 0.005).

**Table 3:** Risk of an incident hospital diagnosis of depression according to the suPAR level.

		HR	95% CI	P-value
**All, n = 9275**	suPAR	1.28	1.08, 1,53	0.005
**Events = 22**	sex	0.56	0.23, 1.35	0.19

#### Previous use of antidepressants and suPAR

Of 9305 donors, 445 purchased antidepressant medicine in the period from January 1995 to the day of suPAR measurement in the beginning of 2006. The majority (54.4%) of those who used antidepressants had three purchases or fewer. Table 4 shows the results from the linear regression analysis with purchase of antidepressants as an explanatory variable. For men, previous use of antidepressants was associated with higher suPAR levels, when the last purchase occurred less than 12 months before suPAR measurement. The suPAR level in men who purchased their antidepressant medication more than 12 months prior to suPAR measurement was not significantly higher than that in men who never purchased antidepressant medicine. For women, suPAR levels were statistically significantly higher in those with previous use of antidepressants in all categories of time from last purchase to suPAR measurement. Women who purchased antidepressant medicine less than six month before suPAR measurement had a log2-transformed suPAR value that was 0.17 (95% CI: 0.03–0.32) higher than women who never used antidepressants. The effect was smaller for women who made their last purchase 6 to 12 months before suPAR measurement (0.06 (95% CI: 0.001–0.11)).

**Table 4:** Effect^1^ of previous use of antidepressants on log2-transformed suPAR.

	Time since last purchase, (months)	Effect on suPAR	95% CI	P-value
**Men, n = 4841**	less than 6	0.30	0.11, 0.50	0.002
	6 to 12	0.22	0.04, 0.40	0.02
	12 or more	0.05	−0.2, 0.13	0.15
**Women, n = 4463**	less than 6	0.17	0.03, 0.32	0.02
	6 to 12	0.16	0.004, 0.32	0.04
	12 or more	0.06	0.001, 0.11	0.045

1 Estimates are from linear regressions adjusting for age (continuous).

Thus, there was a trend towards declining effect on the suPAR level with increasing time because the last antidepressant purchase, although the associations between suPAR level and antidepressant purchase were not significantly different for the three timing categories in women.

## Discussion

To our knowledge, this is the first larger prospective population-based study of the inflammatory marker suPAR and use of antidepressants. We investigated the association of suPAR levels and both subsequent incident use of antidepressants and prior use of antidepressants and found an association between high suPAR levels and the use of antidepressants in both directions.

When analysing the incident use of antidepressants, we found a statistically significant effect of suPAR level on the risk of subsequent use of antidepressants during a five-year observation period. To our knowledge, this is the first larger prospective population-based study of the inflammatory marker suPAR and use of antidepressants. We investigated the association of suPAR levels and both subsequent incident use of antidepressants and prior use of antidepressants and found an association between high suPAR levels and the use of antidepressants in both directions. Kaplan-Meier curves depicting suPAR quartiles indicate that a high suPAR level was associated with an increased risk of antidepressant usage throughout the observation period. This is a surprising and interesting observation, suggesting that an elevated suPAR level seems to reflect a constitutively increased risk of depression.

We also found that prior use of antidepressants was significantly associated with higher suPAR levels in both men and women. This could possibly be an example of reverse causality, as we cannot rule out that the suPAR level was already increased before the purchase of antidepressants. For men, the effect was greater when the purchase was temporally closer to the suPAR measurement. For women, the effect was independent of purchase timing. There was, however, a trend towards a decreased strength of the association when the time between suPAR measurement and the purchase of the antidepressant medicine increased. The timing categories were based on clinical guidelines for treatment in the acute and maintenance phases and are in accordance with prior studies [24], [25].

Our findings are in line with studies of depression and CRP that found an association between CRP levels and prior depression [26]–[28]. Two of these studies, however, only detected the association in men and not in women. In this study, we found an effect for both men and women.

It is well known that there is an increased prevalence of depression among patients with diseases such as diabetes [29], myocardial infarction [30] and cancer [1], [2], [7], [31], [32]. Thus, to examine whether the relationship between suPAR levels and the subsequent use of antidepressants was caused by another underlying disease affecting both suPAR level and depression, we adjusted the analyses for prescription medications other than antidepressants. The purchase of medication, other than antidepressants, was not associated with an increased risk of antidepressant usage and did not affect the relationship between suPAR levels and the incident use of antidepressants. This does not suggests that treatment with antidepressant medicine could have been initiated in connection with an underlying other disease.

This study is for the outcome based on register data using the purchase of antidepressant medicine as a proxy for depression. The same ATC code (N06A) has previously been used in other population-based studies of depression [24], [25], [33], [34]. Whether our use of register data on antidepressant use captures the donor with a depression is subject to discussion. However in Denmark can such medication only be prescribed by physicians and regulation and surveillance of the use of medication is quite strict. The sensitivity of antidepressant use as a proxy for major depression has been evaluated to be up to 50% [35] while, more importantly, specificity seems to be more than 90%.

Thus, it seems reasonable to use the purchase of antidepressant medicine as a proxy for depression, acknowledging that milder forms of depression may not all be recorded. The sensitivity of 50% indicates that many people with depression are not prescribed medication and there can be a long delay between the onset of depression and initiation of the antidepressant use. Further, antidepressants are used for a number of indications other than major depression. Thus, we cannot completely rule out that a proportion of participants who started using antidepressants after the suPAR measurement were already depressed or had been depressed before blood collection without receiving an antidepressant at that time. Also did we in this analysis exclude all donors who had purchased antidepressants prior to the suPAR measurement. However older donors could potentially have been treated with antidepressants before the register started in 1995.

A limitation of our study is the lack of information on relevant potential confounders such as smoking, alcohol consumption, BMI and physical activity. Smoking may lead to increased suPAR levels [36] and is common among depressed patients. Obesity has been suggested to be a potential link between depression and elevated CRP [27], [37]; however, no effect of obesity on depression was found in the 12-year US national health survey [38]. The MONICA cohort study found that BMI and waist circumference were correlated with CRP but not with suPAR [21]. Thus, we cannot exclude that the suPAR level reflects lifestyle factors statistically associated with depression. On the other hand could the same factors also be acting as mediators of the association between high suPAR levels and subsequent use of antidepressants. However, the cohort consists of blood donors who at each donation claim to be healthy, are able to donate blood on a voluntary basis and agree to participate in the study. In several studies blood donors are characterized as being very healthy [39], [40]. Our conclusions are therefore valid in a healthy adult population only.

A second limitation of this study is the lack of a comparison between suPAR measurements and other components of the fibrinolytic system or other inflammatory markers previously shown to be associated with depression, such as CRP or IL-6 [41]. In patients with non-malignant diseases characterised by systemic immune activation, suPAR levels are correlated with TNF-α and TNFrII. Some studies have observed a positive correlation between IL-6 and suPAR level, while others have not [42], [43].

suPAR has not been previously investigated in depression. In neurological diseases such as Alzheimer, multiple sclerosis, HIV dementia, cerebral malaria and Creutzfeldt-Jakob disease, the presence of uPAR in macrophages/microglia within the CNS has been described [44]–[48]. An animal model of cerebral inflammation with intracerebral LPS injections induced uPAR expression in microglia at both the mRNA and protein levels compared to controls [49]. Further studies are needed to investigate the role of uPAR produced in the cerebrum and its association with depression.

We conclude that high suPAR levels are associated with an increased risk of subsequent incident purchase of antidepressants and an incident hospital diagnosis of depression. Conversely, the prior purchase of antidepressants is associated with increased suPAR levels. We are not able to identify methodological drawbacks that clearly refute these findings as artefacts, though we did not have data on a number of important potential confounders. suPAR is an inflammatory marker and high levels could thus reflect a subclinical state of inflammation that increases the vulnerability to depression. Our findings support the theory that low-grade inflammation contributes to the pathogenesis of depression, adding depression to the list of diseases associated with low-grade inflammation. Further studies are needed to characterize the exact role of suPAR in depression in clinical practice.
